# Heparan Sulfate and Sialic Acid in Viral Attachment: Two Sides of the Same Coin?

**DOI:** 10.3390/ijms23179842

**Published:** 2022-08-30

**Authors:** Ivan Emmanuel Ramos-Martínez, Edgar Ramos-Martínez, René Álvaro Segura-Velázquez, Manuel Saavedra-Montañez, Jacquelynne Brenda Cervantes-Torres, Marco Cerbón, Dulce Papy-Garcia, Edgar Zenteno, José Ivan Sánchez-Betancourt

**Affiliations:** 1 Departamento de Medicina y Zootecnia de Cerdos, Facultad de Medicina Veterinaria y Zootecnia, Universidad Nacional Autónoma de México, Ciudad de México 04510, Mexico; 2 Facultad de Química, Universidad Nacional Autónoma de México, Ciudad de México 04510, Mexico; 3 Unidad de Investigación, Facultad de Medicina Veterinaria y Zootecnia, Universidad Nacional Autónoma de México, Ciudad de México 04510, Mexico; 4 Departamento de Microbiología e Inmunología, Facultad de Medicina Veterinaria y Zootecnia, Universidad Nacional Autónoma de México, Ciudad de México 04510, Mexico; 5 Unidad de Investigación en Reproducción Humana, Instituto Nacional de Perinatología-Facultad de Química, Universidad Nacional Autónoma de México, Ciudad de México 04510, Mexico; 6 Glycobiology, Cell Growth ant Tissue Repair Research Unit (Gly-CRRET), Université Paris Est Créteil (UPEC), F-94010 Créteil, France; 7 Departamento de Bioquímica, Facultad de Medicina, Universidad Nacional Autónoma de México, Ciudad de México 04510, Mexico

**Keywords:** Heparan sulfates, sialic acid, glycoconjugates, virus receptors, viral entry

## Abstract

Sialic acids and heparan sulfates make up the outermost part of the cell membrane and the extracellular matrix. Both structures are characterized by being negatively charged, serving as receptors for various pathogens, and are highly expressed in the respiratory and digestive tracts. Numerous viruses use heparan sulfates as receptors to infect cells; in this group are HSV, HPV, and SARS-CoV-2. Other viruses require the cell to express sialic acids, as is the case in influenza A viruses and adenoviruses. This review aims to present, in a general way, the participation of glycoconjugates in viral entry, and therapeutic strategies focused on inhibiting the interaction between the virus and the glycoconjugates. Interestingly, there are few studies that suggest the participation of both glycoconjugates in the viruses addressed here. Considering the biological redundancy that exists between heparan sulfates and sialic acids, we propose that it is important to jointly evaluate and design strategies that contemplate inhibiting the interactions of both glycoconjugates. This approach will allow identifying new receptors and lead to a deeper understanding of interspecies transmission.

## 1. Introduction

Glycosylation is the most abundant post-translational modification; it is estimated that more than 50% of the proteins in the human genome are associated with saccharides, mainly proteins that cross the secretory pathway. Glycoconjugates are an important component of the cell membrane and the extracellular matrix and, therefore, play a relevant role in intercellular interactions [1].

Glycoconjugates are variable in their composition, structure, and localization; characteristics that will determine the so-called “glycan code” or “sugar code”. In turn, this information will depend on the joint action of glycosyltransferases and glycosidases expressed by each cell type [2]. The presence of lectins that recognize carbohydrates in a specific manner can induce multiple responses in physiological and pathological conditions, which can occur among cells themselves or between cells and pathogens like bacteria, parasites, and viruses. We will focus on viruses in this review.

In mammals, the two most common types of glycoconjugates involved in viral recognition are those associated with sialic acids and heparan sulfate proteoglycans. Heparan sulfates (HS) are the glycan part of heparan sulfate proteoglycans (HSPGs) and share similar distribution with sialic acids in the most superficial part of the cell membrane, being important components of the extracellular matrix and having negative charge in physiological conditions, these characteristics allow them to interact electrostatically with basic residues [3].

Herpes simplex virus (HSV), human papillomavirus (HPV), and dengue virus bind directly to HS during the entry process, whereas other viruses occupy HS as co-receptors to facilitate binding to the main receptor or attract the highest number of virions through weak interactions, among these latter viruses are the respiratory syncytial virus, Zika virus, foot-and-mouth disease virus, and SARS-CoV-2 [4]. Among the viruses that occupy sialic acids as a gateway, we can find influenza A viruses, adenoviruses, parvoviruses, etc. (Table 1). Influenza A viruses are a classic example where interspecies transmission can occur by modifying their affinity for sialic acid at different positions [5]. The latter demonstrates the importance of lectin-glycan interaction in zoonotic diseases.

An additional similarity between HS and sialic acids is their high expression in mucosal epithelial cells of the respiratory and gastrointestinal tract, the most common pathogen entryways. Therefore, this work aims to review the information of some viruses where the participation of glycoconjugates in the entry process has been characterized and reports exist that suggest the participation of a second type of glycoconjugate. For example, in HSV-1 the role of HS in cell attachment is widely described; however, some studies suggest the participation of sialic acids in viral infection [6,7]. Another example is the influenza A virus; recently, it has been observed that the presence of HSPG is related to the severity of the disease, in addition to the possibility of inhibiting virus binding in vitro using sulfated oligosaccharides [8,9]. These examples will be described in detail in later sections.

We will also address the different strategies focused on trying to inhibit the interaction of the virus with the glycoconjugates of the target cells, which is a well-studied therapeutic target; however, so far there is no effective therapy that inhibits viral entry. In this example, we mention some molecules that have been evaluated in this context for the different viruses covered in this review, from “classically” used molecules such as heparin and lactoferrin, to new strategies such as the use of mimetic molecules, nanomaterials, and bioinformatics approaches.

**Table 1:** Glycoconjugates involved in the viral entry.

Heparan Sulfate
Herpes Simplex Virus type 1 [10]
Dengue virus [11]
Zika virus [12]
Hepatitis C virus [13]
Rabies virus [14]
Human papillomavirus [15]
SARS-CoV-2 [16]
Cytomegalovirus [17]
**Sialic Acid**
Enterovirus D68 [18]
Influenza A virus [19]
Adenovirus [20]
Human polyomavirus [21]
Reovirus [22]
Porcine rubulavirus [23]
Parainfluenza virus type 3 [24]
Porcine reproductive and respiratory syndrome virus (PRRSV) [25]

## 2. Structure and Function of Heparan Sulfate Proteoglycans

The heparan sulfate proteoglycans (HSPGs) are glycoconjugates present in the cell membrane and the extracellular matrix, their basic structure consists of a core protein and a heparan sulfate chain (HS), a type of glycosaminoglycan formed of anionic saccharides. HSPGs are widely expressed in the organism and can be found as membrane proteins, such as the syndecans; anchored to the membrane by glycosylphosphatidylinositol, like the glypicans; or secreted into the extracellular space to be part of the extracellular matrix, as in the case of perlecan and type XVIII collagen. In addition, HSPGs are involved in virtually all processes related to intercellular communication such as proliferation, angiogenesis, and inflammation, in addition to various pathologies such as cancer and neurodegenerative diseases [26,27,28]. The variability and part of the functions of HSPGs are determined by the heparan sulfate chain that is synthesized by the set of enzymes present in each cell type.

**Figure 1:**
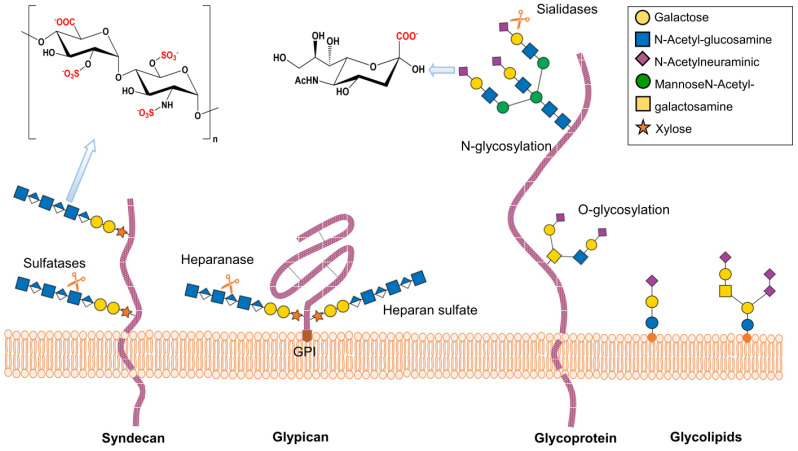
Schematic structure of heparan sulfate proteoglycan and sialic acid, the most important glycoconjugates that serve as viral receptors.

The synthesis of HS begins at specific serine residues, where xylose-galactose-galactose-galactose-glucuronic acids are sequentially added; then, elongation begins with repeated units of the *N*-acetylglucosamine (GlcNAc) disaccharide and glucuronic acid (GlcA) forming extensive chains that subsequently undergo various modifications that will depend on the set of enzymes present in the RER-Golgi network (Figure 1). We will explain, in general terms, the modifications carried out by these groups of enzymes during the maturation process of the HS chains: the first group of four isoforms of deacetylase/N-sulfotransferases 1–4 (NDSTs 1–4) are in charge of changing the N-acetyl groups by N-sulfate groups in GlcNAc, subsequently, the Glucuronyl C5-epimerase carries out the conversion of D-GlcA to its epimer L-IdoA. Finally, a set of O-sulfotransferases (OST) adds sulfate groups to glucosamine, the addition at carbon 6 occurs by the action of 6-*O*-sulfotransferases (isoforms 1–3), whereas at carbon 3 it occurs by the action of the group of 3-*O*-sulfotransferases (isoform 1–6) [29].

The 3OSTs family is the most numerous family, with seven isoforms: 3OST-1, -2, -3a, -3b, -4, -5, and -6. Each isoform has a specific localization at the tissue level, being the group of enzymes that most contributes to the HS variability, for example, 3OST2, is mainly expressed in the nervous system whereas 3OST6 is mainly expressed in the skin, leading to the formation of different structural domains [30]. In addition, the activity of each 3OSTs isoform will depend on previous modifications made by NDSTs and glucuronyl C5-epimerase, which is reflected as stereochemical changes in the HS chain and the ability to interact with other proteins [31]. The large number of enzymes involved in the synthesis of HS and their selective expression confer a great heterogeneity to HS chains so that each anatomical region will present a different microenvironment depending on the set of enzymes expressed.

Heparin has been a useful tool to study heparan sulfates, its structure is similar but more sulfated, which allows the identification of heparan sulfate binding sites, although classically it has been used as an anticoagulant it also has anti-inflammatory and antimetastatic properties [32].

## 3. Structure and Function of Sialic Acid

The family of sialic acids are a group of 9-carbon monosaccharides that include more than 80 compounds where N-acetyl neuraminic acid (Neu5Ac) is the most common in mammals [33]. Neu5Ac is modified by O-acetylation, O-sulfation, O-methylation, O-lactylation, or O-phosphorylation at its hydroxyl groups at carbons 4, 7, 8, or 9 [34]. The hydroxylation of the N-acetyl group of Neu5Ac gives rise to N-glycolylneuraminic acid, another commonly found sialic acid in animals [34]. Some sialylated proteins serve as entry receptors for viruses and bacteria, as in the case of integrins and mucin-like glycoproteins. Additionally, sialylated gangliosides can also serve as receptors for pathogens [5]. 

Sialic acids can bind to *N*-acetyl-galactosamine (GalNAc) or N-acetyl-glucosamine (GlcNAc), in both N- and O-glycans, via α-2,3 or α-2,6 linkages. Sialic acid is also found in glycosphingolipids and the side chains of glycosylphosphatidylinositol anchors [35] (Figure 1). These bonds are generated by sialyltransferases, enzymes found mainly in the trans-Golgi network. Differential expression of sialyltransferases generates variability in the sialoglycans present in specific tissues; this difference not only occurs between organisms and cells of different tissues, but also varies concerning time, space, and environmental cues [3].

Another type of sialic acid (SA) compound is polysialic acid, a polymer of up to 100 SA units found in some proteins such as the neural cell adhesion molecule (NCAM) and the synaptic cell adhesion molecule 1 (SynCAM 1); in addition, it is present in some pathogenic bacteria [3], where SA is bound by α-2,8 or α-2,9 bonds [36]. 

Sialidases or neuraminidases are enzymes that cleave the bonds that hold sialic acids to glycans. Many microorganisms display sialidases that remove sialic acid to facilitate their interaction with their receptors [37]. Some sialidases are very specific, *Streptococcus pneumoniae* sialidase acts on sialic acid with α2-3 bonds, whereas others have a broad spectrum of activity such as the *Arthrobacter ureafaciens* sialidase that cleaves sialic acid bound with α2-3, α2-6, or α2-8 bonds [3].

## 4. Herpes Simplex Virus

HSV-1 and HSV-2 from the subfamily Alphaherpesvirinae are double-stranded DNA and enveloped viruses that cause mucocutaneous lesions and can infect epithelial cells, neurons and, more rarely, fibroblasts. It is estimated that more than half of the world’s population is infected with HSVs, the infection remains throughout life accompanied by sporadic reactivations that are usually asymptomatic, representing a significant burden for health services and the global economy [38].

### 4.1. HSV Receptors

The entry process involves several viral glycoproteins that follow sequential steps, starting with the attachment of the virus to the cell by the glycoprotein D (gD) that can recognize three different receptors from the cell membrane, nectin-1, herpesvirus entry mediator (HVEM), and 3-O-sulfated heparan sulfate (3-OST HS) [10,39]. The interaction of gD with its receptors on the cell surface allows the subsequent formation of a complex with the gH-gL heterodimer that ultimately leads to the activation of glycoprotein B (gB), which is the main component of the fusion pore between the cell membrane and the viral envelope [40].

The three receptors described for HSVs interact with gD at distinct but overlapping sites, as shown by the analysis of amino acid substitutions and deletions; additionally, the N-terminal end of gD is required for binding to HVEM and 3-OST HS, but not for the binding of nectin-1 [41]. The relevance of heparan sulfate in the interaction of HSV type 1 and 2 with cells was first described by WunDunn and Spear [7]; subsequently, it was observed that gD recognizes more efficiently the 3-O-sulfated form of heparan sulfate [10]. As discussed above, 3-O-sulfation is synthesized by a family of six enzyme isoforms (3-OST-1, 3-OST-2, 3-OST-3A, 3-OST-3B, 3-OST-4, and 3-OST-5), which synthesize different 3-O-sulfated motifs and are expressed in an organ-dependent manner. Changes in the expression of these enzymes lead to the alteration of their binding capacity to their natural receptors, as observed in pathologies such as cancer and neurodegeneration [42,43].

Most 3-OSTs generate receptors for gD [44,45,46] except 3-OST-1, which does not generate HS chains capable of interacting with virions; this was tested using CHO cells and zebrafish expressing only 3-OST-1 that were resistant to viral infection [47].

Bacsa et al. (2011) performed downregulation studies of syndecan-1 and syndecan-2, which are proteoglycan heparan sulfates widely expressed in humans; these authors observed that siRNA silencing of syndecan-2 significantly reduces HSV-1 infection, however, this effect was less when syndecan-1 was silenced. They explained this result as being due to the fact that syndecan-2 is rich in heparan sulfates and syndecan-1 contains chondroitin sulfates and heparan sulfates. Additionally, they found that 2 h post-infection with HSV-1 increases the expression of syndecan-1 and syndecan-2 [48]. Subsequently, it was found that the post-infection increase in heparan sulfate expression is transient as HSV-1 positively regulates heparanase (HPSE) to remove heparan sulfates allowing virus release, whereas knockdown of HPSE drastically decreases its release [49]. Furthermore, there is evidence that the knockdown of the syndecan-1 ectodomain by HPSE action is important for HSV-1 release [50]. This suggests that HSs are not only involved in the entry process of HSVs but also in the process of virus release and spread.

The relevance of heparan sulfates in HSV-1 entry has been suggested by several competitive and enzymatic assays that remove heparan sulfates from the cell membrane. Such is the case of the inhibitory effect of heparin on HSV infection in leukocyte cultures [51], the use of cationic peptides that bind heparan sulfate to inhibit HSV-2 entry in cell culture, and in mouse models of HSV-2 infection [52].

Enzyme treatment with heparinase in primary cultures of human corneal stromal-derived fibroblasts blocked viral infection. The authors attribute this effect to the fact that incubation with heparinase II and III for 90 min was sufficient to remove heparan sulfates from the cell surface; interestingly, the same enzyme treatment in nectin-1-expressing HeLa cells only partially decreased infection after heparinase treatment [53].

The use of synthetic 3-O-sulfonated heparan sulfate to block viral infection of Vero cells has been explored therapeutically, showing efficacy of up to 80% inhibition at micromolar concentrations [54]. The use of glycosaminoglycan mimetics as potential therapeutic agents capable of inhibiting the interaction of HSVs with cells has also been tested. Gangji et al. tested the effect of glycosaminoglycan mimetics (NSGM) with different structures such as flavonoid-based, benzofuran-based, and glycoside-based. After infecting different cell lines with HSV-1 they found that glycoside-based NSGMs are efficient inhibitors of HSV-1 entry [55]. Cacicol^®^, a biomimetic heparan sulfate used in regeneration therapy was able to inhibit viral infection in Vero and Hep-2 cell lines; therefore, the authors attribute antiviral activity and potential therapy to it [56]. The currently deployed drugs against HSVs are focused on inhibiting viral DNA replication as is the case of acyclovir. However, the development of new treatments focused on inhibiting viral entry, such as glycosaminoglycan mimetics, is currently in progress.

### 4.2. HSV and Sialic Acids

Some studies suggest that sialic acid may play an active role during HSV-1 infection since gD and gB glycoproteins are sialylated. Interestingly, Teuton and Brandt found that virions subjected to neuraminidase digestion decreased titers up to 100-fold and that sialic acid-specific lectins interfere with infectivity, suggesting that alpha2,6-linked sialic acids are necessary for infection and play an important role between cell binding and internalization into the nucleus [57]. Subsequently, it was found that MAG (myelin-associated glycoprotein), a member of the sialic-acid-binding Ig-like lectin (SIGLEC) family, is necessary for the virus entry process and can bind to gB [6]. To be considered also is that lactoferrin, a highly sialylated glycoprotein, can inhibit HSV-1 infection in vitro by blocking virus entry into the cell [58]. These observations support the theory proposed by Teuton and Brandt that sialic acids of viral proteins are necessary for infection and that could be recognized by cell membrane lectins. So far, an entry pathway involving sialic acids has not yet been described. However, these interactions may contribute to creating a microenvironment that favors the binding of the virus to its final receptors and should be considered as a possible therapeutic target against HSVs.

## 5. Influenza A Virus

Influenza viruses (IAVs) are enveloped viruses with a negative-sense single-stranded RNA genome that belong to the Orthomyxoviridae family, which includes the influenza A, B, C, and D genera. Influenza A is the most virulent type for humans and is a zoonotic disease that affects a large number of species [59,60]. Therefore, this review will focus on the influenza A virus (IAV) only.

Hemagglutinin (HA) and neuraminidase (NA) are the most abundant proteins in the envelope and so far, 18 HA and 11 NA types are known. The combination of these proteins allows classifying IAVs into different subtypes that are species-characteristic: HA1-16 and NA1-9 have been found in birds, HA17-18 and NA10-11 have been isolated from bats, and in humans, we can generally find HA1-3 and 5 [61,62,63].

### 5.1. IAV Receptors

The HA of IAVs is the main protein responsible for cell surface binding, which recognizes sialic acids from cell membrane glycoproteins. Generally, α2,6-linked sialic acid is recognized by strains infecting the human respiratory tract and α2,3-linked sialic acid is recognized by avian viruses [19,64]. Species jumping is rare due to molecular, physiological, and spatial barriers. However, given the high mutation rate, it is likely to occur resulting in outbreaks with pandemic potential [65]. Recently, it has been described that HA can bind to other receptors, such as phosphorylated N-glycans in the human lung [66], or MHC class II molecules in a glycan-independent manner [67].

The interaction of HA with sialic acids has been a therapeutic target for the development of molecules capable of inhibiting viral recognition of their natural receptors. Different sialic acid derivatives, such as sialylphosphatidylethanolamine [68], gold nanoparticles with sialic acid terminated [69], sialic acid conjugated with pentacyclic triterpene [70], etc., have been tested for several years. All these molecules have shown effectiveness in vitro and in hemagglutination inhibition assays. Peptides derived from bovine lactoferrin have also been used and were able to inhibit infection in in vitro assays [71]. Other options that have been tested are sialic acid-mimic peptides, which resemble cell surface glycoproteins [72], and fluorinated analogues of sialic acid; the latter has shown a much higher affinity for HA compared to sialic acid. In addition, the latter has the peculiarity of escaping the action of neuraminidase [73].

Computational methods have contributed to optimizing the search for HA blockers, allowing for selection among thousands of compounds theoretically and the evaluation of the best candidates experimentally. Some molecules chosen by this methodology are the N-benzyl-4,4,-disubstituted piperidines [74] and the derivatives of compound NSC8556 [75]; both showed in vitro effectiveness to inhibit virus entry. So far, the only drugs used against influenza are neuraminidase inhibitors such as oseltamivir. The strategy of blocking HA interaction with cell membrane sialic acids has only been effective in in vitro experiments, translating these results to in vivo models represents a great challenge given the complexity of the physiological microenvironment and potential interactions.

### 5.2. IAV and Heparan Sulfates

Recently, a study has associated the components of the glycocalyx with the prognosis of influenza A H1N1, finding that plasma levels of syndecan-1, hyaluronan, and heparan sulfate are elevated in patients with severe influenza relative to mild cases, and in the case of syndecan-1, are strongly correlated with mortality [76]. In contrast, in a previous work using a syndecan-1 −/− mice model a protective role was attributed when syndecan-1 was associated with bronchial epithelium during influenza A H1N1 infection, decreasing inflammation and limiting apoptosis [8]. In a mouse model, CD11c+ cells were silenced for NDST1 to evaluate the role of heparan sulfates in the immune response to influenza A infection and the authors found decreased inflammation, increased IFN-β expression, and overall a more optimal response to virus clearance [77]. The authors of these studies suggest that although the role of heparan sulfate and proteoglycans is unclear, they could be a target for the design of new strategies against IAVs. It appears that the localization of heparan sulfate in the context of infection is important, and its presence may have opposing effects depending on the organ and cell type. 

Heparin derivatives with low coagulant activity showed the ability to inhibit H5N1 influenza virus infection using in vitro experiments [78]. Sulfated compounds such as sulfated oligofucosides that bind to HA, H1, and H3 have also been tested and shown to be effective in blocking virus entry in cell cultures using MDCK cells [79]. Inhibition of infection in vivo and in vitro was achieved using sulfated chitooligosaccharides; furthermore, the authors demonstrated that sulfation of chitooligosaccharides is essential for antiviral activity [9].

## 6. SARS-CoV-2

SARS-CoV-2 is a positive-sense RNA-enveloped virus of the genus Betacoronavirus, which has been a pandemic since 2019. It has a zoonotic origin suspected to have arisen from wild mammals and, so far, continues cross-infection with other mammals, which is a potential source of new outbreaks [80].

The virion envelope contains the spike protein, which is involved in binding to the cell surface and the fusion process. The S protein has two subunits, the S1 subunit that interacts with ACE2 as its main receptor, and the S2 subunit that is involved in fusion with the host cell [81]. The N-terminal end of protein S has several N-glycosylations, although it is unknown whether this plays a role in the infection process the fact that some neutralizing antibodies are directed to the N-terminus suggests that it has an important role [82]. Another receptor described is the transmembrane serine protease 2 (TMPRSS2) [83].

### 6.1. SARS-CoV-2 Receptors

The S1 subunit of SARS-CoV-2 can bind heparin and heparan sulfate at a site other than the ACE2 binding site, so that respiratory tract heparan sulfates may act as a co-receptor favoring binding to other receptors [16]. In vitro studies showed that spike protein has a preference for 3-O-sulfated heparan sulfate, mainly chains generated by 3OST-3B isoform; however, this work has not been peer-reviewed [84]. In addition, neutralizing antibodies isolated from patients with COVID-19 have been observed to interfere with the virus binding to heparan sulfates [85]. In vitro experiments with the Delta variant showed that syndecan-4 and syndecan-1 facilitate the binding of the virus to the cell surface, with syndecan-4 having a greater effect [86]. Interestingly, the authors suggest that the greater presence of basic amino acids in the spike proteins of the Delta variant favors the interaction with the negative charges of HS, resulting in a greater affinity that could explain its greater propagation capacity.

Considering the role of HS in viral binding, sulfated polysaccharides derived from Saccharina japonica, sulfated galactofucan, and glucuronomannan were studied by surface plasmon resonance, which showed a strong binding ability to SARS-CoV-2 protein S [87]. In another study, a large number of sulfated glycans were tested, among which pentosan polysulfate (PPS) and mucopolysaccharide polysulfate (MPS), evaluated by surface plasmon resonance, showed the strongest binding ability. Subsequently, these two compounds were evaluated in vitro by infecting cultures of Vero cells. Both compounds showed a greater capacity to inhibit viral infection than heparin, another independent group also evaluated the effectiveness of pentosan polysulfate in vitro using HEK293T cells, obtaining similar results [88,89].

Another alternative that has been explored is the use of heparin polysaccharide nanoparticles as nanodecoy. This strategy was tested in vivo using a SARS-CoV-2 pseudovirus infection model in BALB/c mice, where nasal administration in the first 24 h is effective in reducing lung infection [90].

An alternative approach is the use of the cationic peptides G1 (LRSRTKIIRIRH) and G2 (MPRRRRRRRRRRQK) as a prophylactic treatment, which revealed in silico binding affinity studies that they can bind HS and in vitro studies showed that they are able to compete with the virus and decrease its binding to cell surface HS [91].

### 6.2. SARS-CoV-2 and Sialic Acids

There are reports that the spike protein can recognize structures with sialic acid, as shown in a study where saturation transfer difference nuclear magnetic resonance (STD NMR) was used revealing that the N-terminal domain is capable of interacting with α2,3 sialyl N-acetyllactosamine and α2,6 sialyl N-acetyllactosamine trisaccharides, with preference when the sialic acid is bound by the α2,3 bond [92]. In another study, it was observed that protein S binds sialylated gangliosides with a similar affinity with which it recognizes HS. Furthermore, treatment with neuraminidase and genetic deletion of sialyltransferases have a negative effect on viral entry [93]. Considering that STD NMR is one of the most reliable techniques to evaluate the interaction between molecules and the effect of sialic acid deletion, we can infer that sialic acids are important co-receptors favoring SARS-CoV-2 binding. 

However, contrasting results were observed in a study using human lung and intestine tissues ex vivo, where ACE2 sialic acids were found to restrict binding to spike protein; furthermore, treatment with neuraminidase or mutant cells with decreased expression of sialic acids favored the binding of ACE2 to protein S [94]. In a similar work, where cells were treated with sialidases to cleave sialic acids at the different α2-3,6,8 positions, the removal of α2-6 sialic acid slightly decreased the Kd of protein S by ACE2, whereas the use of other glycosidases such as α-fucosidase had no significant effect [95]. Although these works support the theory that the presence of sialic acids decreases the binding of the virus to ACE2, the fact that only sialic acid in position α2-6 has this effect leads us to propose that there is specificity for the binding of sialic acids to galactose.

Viral envelope proteins are often glycosylated, as is the case of spike protein which is rich in N-glycans and O-glycans terminating in sialylation or fucosylation [96]. There is evidence of endogenous lectins present in the cell membrane capable of interacting with virus envelope glycoproteins (Table 1). For example, it has been reported that sialic acid-binding Ig-like lectin 1 (Siglec-1/CD169) allows antigen-presenting cells such as dendritic cells to bind SARS-CoV-2 [97]. In another work, it was observed that the virus can interact with other endogenous lectins such as C-type lectin receptors, DC-SIGN, and L-SIGN, which contribute to pathogenesis by enhancing ACE2-mediated infection [98].

The binding of SARS-CoV-2 to heparan sulfates and sialic acid-containing glycans has been explored by Hao et al. (2021). Their results revealed that spike proteins bind to heparan sulfate but not sialic acid. This type of approach where both glycoconjugates are evaluated will allow us to better understand if there is a synergistic relationship with viral infection [99].

## 7. Human Papillomavirus

Human papillomavirus (HPV) is the most common sexually transmitted virus in the world and is one of the main causes of cancer; it possesses a double DNA strand and lacks an envelope. Effective HPV vaccines are currently available, however, they are expensive and do not protect against all types of HPV [100]. HPVs infect epithelial tissue; however, each type of HPV has tropism to specific organs. For example, HPV6 mainly infects genital epithelium, HPV7 causes lesions on the soles of the feet and hands, HPV11 affects the oral cavity, etc. This high specificity of HPV types for their target organs suggests that the microenvironment where the infection develops is important [101,102].

### 7.1. HPV Receptors

The capsid of HPVs is mainly composed of the L1 protein, which can be recognized by several receptors and confers alternative pathways for cell entry [103]. Although there is no mandatory receptor for HPV entry, host heparan sulfates are essential factors that provide the microenvironment necessary for the initial attachment of virions, either by direct binding to the cell surface through syndecan-1 or by transient binding to laminin-5 of the extracellular matrix. Therefore, HPVs remain bound to the extracellular matrix for hours until they infect proliferating keratinocytes [15,104]. This feature is known as asynchronous entry, which is very particular to HPVs, where some virions can enter the cell within minutes whereas others can take hours to initiate the entry process [105].

Heparin and carrageenan, a sulfated polysaccharide, were found to bind to HPV capsids and block the infectivity of cultured CHO-K1 and pggs-745 cells. Treatment with carrageenan had a three-orders of magnitude more potent effect than heparin [106]. A similar result was observed when using Escherichia coli K5 polysaccharide with a high degree of O-sulfation that prevented in vitro infection of cells by HPV-16, HPV-18, and HPV-6 pseudovirions. When unmodified E. coli K5 polysaccharide and N-sulfated E. coli K5 polysaccharide were tested, the inhibitory effect was not obtained, demonstrating the importance of O-sulfation [107]. Recently it was observed that protamine sulfate, a heparin neutralizing drug, inhibits the in vitro infection and the mouse model of infection of several types of HPVs, the authors suggest that these results indicate that blocking the interaction of the virus with the heparan sulfate of the cells could be a prophylactic treatment to prevent infection [77].

### 7.2. HPV and Sialic Acids

In in vitro experiments, lactoferrin, a highly sialylated glycoprotein, was tested to inhibit HaCat cell infection; bovine lactoferrin, which has more potential glycosylation sites, had a more potent inhibitory effect than human lactoferrin [108,109]. In an interesting study focused on exploring the vaginal microbiota and enzymes produced during different bacterial vaginosis, authors found that Gardnerella vaginalis infection was a higher risk factor for HPV than other bacteria, and G. vaginalis produces sialidase, an enzyme that cleaves sialic acids. The authors suggest that sialidase is the key factor facilitating HPV infection in cervical lesions [110]. 

So far, there is no clear evidence suggesting the involvement of sialic acids as HPV receptors; however, it would be interesting to evaluate the combined use of lactoferrin with heparan sulfate mimetics, considering the above-mentioned results.

## 8. Adenovirus

The adenoviridae family includes more than 100 serotypes that can infect many vertebrate groups. Of these serotypes, about 50 infect humans and have been grouped into seven species named A through G [111]. Adenoviruses can cause respiratory, ocular, urinary tract, and gastrointestinal infections [112]. Adenovirus infections are highly contagious and can cause local epidemics [111].

### 8.1. Adenovirus Receptors

The capsid of adenoviruses possesses a fibrillar protein, which contains a globular C-terminal domain known as a “knob” that mediates the initial interaction with its receptors, such as the coxsackie virus and adenovirus receptor (CAR), CD46, desmoglein-2, GD1a glycan, and polysialic acid [113,114]. After the binding of the capsid fibers to their receptor, the proteins begin to depolymerize allowing the binding of another protein, known as penton base, to an integrin; mediating entry into the cell by endocytosis [113]. The interaction of adenovirus with other receptors has been extensively described in other reviews [113]; here, we will focus on glycan-containing receptors.

Adenovirus 52, a member of group G, has two different fibers, a long and a short one. The long fiber binds to CAR and the short fiber binds to a polysialic acid [115]. Adenovirus 37 and 19p have been shown to infect fiber knobs at three sialic acid binding sites, bound in α(2,3)- or α(2,6) position [116]. 

Adenoviruses can recognize sialic acids bound to lipids as well as proteins. For example, ganglioside GD1 has two terminal sialic acids that can be recognized by adenovirus knob fibers [117] and turkey adenovirus 3 uses N-glycoprotein sialic acids for binding to B cells [118]. In addition to the sialic acid binding sites on knob fibers, some adenoviruses have other sialic acid binding motifs, such as the canine adenovirus 2 that has Siglecs (sialic-acid-binding immunoglobulin-like lectins)-type binding motif, which recognizes the Neu5Acα2-3[6S]Galβ1-4GlcNAc epitope [119]. The affinity for its different receptors varies in different types of adenoviruses. For example, adenovirus D26 does not use CD46 or desmoglein-2 as entry receptors and has a low affinity for CAR; its main entry receptor is glycans with sialic acid [120]. On the other hand, adenovirus type 52 uses both CAR and sialylated glycoproteins [20]. The relevance of sialic acid as an entry receptor will depend on the type of adenovirus; some require sialic acid necessarily, in others it is a co-receptor, and in a third group, sialic acid is not required. The design of drugs that inhibit the binding of viruses to their cellular receptors is an interesting avenue for antiviral development. The advantages of this approach are that the first step of viral infection is blocked and the treatment effect is extracellular, which reduces alterations at the intracellular level [121]. Conjugates of 3′-sialylactose with human serum albumin have been proven to inhibit infection of human corneal epithelial cells by adenovirus type 37 [122]. Furthermore, trivalent sialic acid derivatives are potent inhibitors of infection by this adenovirus [123]. Treatment with ME0462, a sialic acid-based molecule, binds to sialic acid binding sites on the knob fibers of adenovirus D 37, D53, and D64 and prevents infection of human corneal epithelial cells [121]. Pentavalent sialic acid conjugates are effective in preventing the binding and infection of human corneal epithelial cells by the human adenovirus 37 and the coxsackievirus A24 variant [124]. Since viruses have multiple fibers to bind sialic acid, it is suggested that multivalent compounds will be more effective in inhibiting adenovirus infection [122,125].

### 8.2. Adenovirus and Heparan Sulfates

Heparan sulfate-proteoglycans (HSPGs) are co-receptors for the binding of several adenovirus serotypes such as serotypes 5 and 2, as heparin treatment reduces the rate of infection of human alveolar type II-derived carcinoma A549 cells by these viruses [126]. The ability of serotype 5 to bind to the coxsackievirus and eliminate the Ad receptor (CAR) suggests that heparan sulfate-proteoglycans are sufficient to mediate its infective capacity in vitro [127]. Similarly, when heparan sulfate expression is suppressed in mouse hepatocytes, the adenovirus 5 is able to infect these cells [128]. Therefore, the virus can opt for different receptors to recognize its target cells. 

Adenovirus subgroup B serotypes 3 and 35 and group D serotype 37 also use heparan sulfate proteoglycans (HS-PGs) as co-receptors for infection [129,130]. Serotype 3 uses the fiber knob domain (knob) to bind to heparan sulfate proteoglycans (HS-PGs), but this is a low-affinity co-receptor and is not the main receptor for this serotype [130] as it is not required to infect lung cells [131]. The mouse adenovirus type 1 (MAV-1) uses heparan sulfate proteoglycans (HSPGs) as primary binding receptors, and this binding depends on N-sulfation and 6-O-sulfation of heparan sulfate proteoglycans [132,133]. Some adenoviruses can interact with the heparan sulfates in coagulation factors. For example, adenovirus 5 and 31 bind to the heparan sulfates of coagulation factors IX and X, which increases their binding and infection of epithelial cells [134]. It has been proposed that the presence of sulfated glycosaminoglycans in secretions or cell membranes is a defense mechanism that prevents or delays virus binding to sialic acid-containing receptors [129].

## 9. Conclusions and Perspectives

A significant proportion of emerging viral infections affecting humans are of zoonotic origin, and a key factor in understanding the spread from one species to another is the affinity of the virus for shared structures that determine the recognition of molecules expressed on the cell surface [135]. Since sialic acids and heparan sulfate are widely distributed in mammals, they represent ideal niches for interspecies jumping and the emergence of pandemic outbreaks.

**Figure 2:**
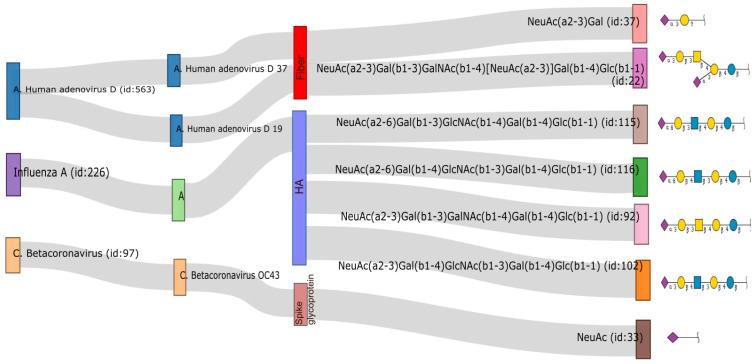
The output of SugarBind Database shows known carbohydrate sequences that contain sialic acid recognized by viruses reviewed in this publication. The representation of glycan in SNFG nomenclature was obtained from the GlyToucan repository.

The heterogeneity of glycosylation has been a constant challenge in the study of protein-glycan interaction. However, viruses can interpret the “glycan language” and recognize these subtle differences in structure, as is the case of influenza A viruses that can discern between α2,3- and α2,6-linked sialic acid [64], or HSVs that have a preference for 3-O-sulfated heparan sulfate except for those generated by the 3-OST-1 isoform [47]. The development of glycomics databases and glycobioinformatics has provided valuable tools for a better understanding of glycan-lectin interactions between viruses and host cells, allowing for the establishment of glycosylation signatures recognized by different types or even strains of viruses (Figure 2), as well as making more accessible information on the physicochemical properties of these glycans and improving the design of new inhibitors [136].

This review did not address lectin–glycan interactions that are part of the antiviral machinery of the immune response such as ficolin, mannose-binding lectin (MBL), and surfactant protein A [137], which are involved in opsonization or antigen presentation processes and generally contribute to the defense against viruses. Rather, it is focused on interactions that are exploited by viruses to increase the affinity for their target cells, leading to a higher infectious capacity.

A large number of molecules have shown effectiveness in inhibiting viral entry in in vitro assays, even molecules such as heparin and lactoferrin have been efficient in blocking the entry of different viruses; however, in most cases it has not been possible to transfer these results to animal models due to the complexity of the physiological context. Among all the variables to consider during in vivo infection, we wish to emphasize the sialic acid/heparan sulfate duality, given the shared characteristics of sialic acids and heparan sulfates. These similarities demonstrate to some extent a biological redundancy, which could be exploited by viruses to infect their target cells more efficiently.

An interesting case is epigallocatechin gallate (EGCG), which can competitively block the primary binding of virions that bind heparan sulfate or sialic acid [138]. Like heparin and lactoferrin, EGCG interferes with the binding of several viruses that are phylogenetically unrelated and possess affinities for different glycoconjugates, so it is of interest to extend knowledge about the interaction between these molecules and their ligands using techniques such as the glycan microarray assay to differentiate affinities between structurally related glycans and saturation transfer difference nuclear magnetic resonance (STD NMR) to identify the epitopes of the glycans [139].

As previously reviewed, some viruses can recognize glycoconjugates to which they bind in a “classical” manner and may also have alternative receptors, as is the case of SARS-CoV-2 and HSV-1, where protein S and protein gB, respectively, can be recognized by cell membrane lectins belonging to the SIGLECs family, which in turn are able to recognize the sialic acids of viral glycoproteins [6,97]. This could partially explain the failure of therapies focused on inhibiting binding to only one type of glycoconjugate, therefore, it would be of great interest to design strategies focused on the simultaneous inhibition of the virus interaction with both glycoconjugates.

## Data Availability

Not applicable.
